# Calf circumference as a screening tool for low skeletal muscle mass: Cut-off values in independent Thai older adults

**DOI:** 10.1186/s12877-023-04543-4

**Published:** 2023-12-08

**Authors:** Jirapa Champaiboon, Aisawan Petchlorlian, Bhorn-ake Manasvanich, Nattaphon Ubonsutvanich, Weerachai Jitpugdee, Piyawan Kittiskulnam, Supharada Wongwatthananart, Yupaporn Menorngwa, Sasitorn Pornsalnuwat, Kearkiat Praditpornsilpa

**Affiliations:** 1 Department of Rehabilitation Medicine, Faculty of Medicine, Chulalongkorn University, Bangkok, Thailand; 2 Geriatric Excellence Centre, King Chulalongkorn Memorial Hospital, Thai Red Cross Society, Bangkok, Thailand; 3 Division of Geriatric Medicine, Department of Medicine, Faculty of Medicine, Chulalongkorn University, Bangkok, Thailand; 4 Department of Family Medicine, King Chulalongkorn Memorial Hospital, Thai Red Cross Society, Bangkok, Thailand; 5 Department of Rehabilitation Medicine, King Chulalongkorn Memorial Hospital, Thai Red Cross Society, Bangkok, Thailand; 6 Department of Medicine, Faculty of Medicine, Chulalongkorn University, Bangkok, Thailand; 7 Department of Nursing, King Chulalongkorn Memorial Hospital, Bangkok, Thailand

**Keywords:** Calf circumference, Sarcopenia, Low muscle mass, Older adults

## Abstract

**Background:**

Calf circumference is recommended as a marker for low muscle mass and as a case finding in the diagnosis of sarcopenia. However, the cut-off value differed by ethic and region. Currently there is no study among Thai population. Therefore, we aimed to identify the optimal cutoff value of calf circumference as a screening tool for low skeletal muscle mass in independent Thai older adults. Subgroup analysis was performed for obesity and adults over 75 years.

**Methods:**

This cross-sectional cohort studied in an outpatient geriatric check-up clinic. Participants, aged 60 and above, needed to be independent in basic activities of daily living to meet the inclusion criteria. Exclusion criteria comprised active malignancy, cardiac, pulmonary, or neurovascular diseases necessitating hospitalization in the preceding three months, chronic renal diseases requiring renal replacement therapy, and unstable psychiatric disorders. We measured the maximum calf circumference and appendicular skeletal muscle mass (ASMI) using bioelectrical impedance analysis (BIA). Low muscle mass is defined according to the Asian Working Group of Sarcopenia (AWGS) 2019 consensus.

**Results:**

We enrolled 6,404 elderly adults (mean age 67.3 ± 5.1 years), with a 47% prevalence of low muscle mass in women and 25% in men. Lower muscle mass significantly correlated with reduced BMI and waist circumference in both genders (p < 0.001). Optimal cut-off values for low muscle mass screening were < 33 cm (sensitivity 80.1%, specificity 60.5%) for women and < 34 cm (sensitivity 85.4%, specificity 70.2%) for men. Subgroup analysis for those with BMI ≥ 25 kg/m² suggested raising the cut-off for women to < 34 cm (sensitivity 80.6%, specificity 54.0%) and for men to < 35 cm (sensitivity 88.7%, specificity 55.2%) to enhance specificity without substantial sensitivity loss. In the older-old adult subgroup (≥ 75 years), optimal cut-off values were < 33 cm (sensitivity 84.6%, specificity 79.9%) for women and < 34 cm (sensitivity 75.6%, specificity 87.0%) for men.

**Conclusions:**

There is a strong correlation between calf circumference and ASMI in independent Thai older adults. Calf circumference can serve as a screening tool for identifying low muscle mass. The recommended cut-off values for men and women are 34 cm and 33 cm, respectively in alignment with AWGS 2019 recommendation. Incorporating a 1-cm higher cut-off value for obese older adults improves the accuracy of muscle mass screening.

**Trial registration:**

Thai clinical trial registry: TCTR20200511003.

## Introduction

Sarcopenia is an age-related generalized skeletal muscle disorder characterized by low muscle mass and low muscle strength or performance that occurs as a natural part of aging and is a multifactorial condition influenced by various factors such as hormonal changes, chronic diseases, malnutrition, physical inactivity, and genetic factors [1]. Sarcopenia has significant health implications, including increased risk of falls, disability, hospitalization, and mortality [2–4]. It also has a negative impact on quality of life and functional independence in older adults [5].

Sarcopenia is typically diagnosed through a combination of physical examination, body composition analysis, and functional tests. Physical examination includes assessing muscle mass, strength, and physical performance, as well as checking for signs of muscle wasting or weakness. Body composition analysis involves measuring muscle mass, and fat mass [6]. The gold standard of muscle mass measurement is magnetic resonance imaging or computed tomography [7]. Dual-energy X-ray absorptiometry (DXA) and bioelectrical impedance analysis (BIA) are more widely used in the clinical setting [8]. Functional tests evaluate a person’s ability to perform activities of daily living, such as walking, climbing stairs, and getting up from a chair [9].

In limited resource settings, where DXA or BIA are not available, the Asian Working Group of Sarcopenia (AWGS) consensus updated in 2019 recommends using calf circumference as an alternative measure of muscle mass and suggests that a calf circumference of < 34 cm for men and < 33 cm for women can be used as a surrogate marker of low muscle mass [10]. Calf circumference is a simple, non-invasive, and inexpensive measure that can be easily obtained in clinical settings [11]. Recent meta-analysis showed that low calf circumference that detects low muscle mass was associated with a higher risk of mortality and has been shown to have a higher screening ability to identify sarcopenia than other AWGS-recommended screening tools. [12]

The cut-off value for calf circumference to define low muscle mass varies from 30 to 35 cm depending on the studied population [13–16]. AWGS 2019 recommendation cutoff value of < 34 cm for men and < 33 cm for women is derived from East Asian countries namely, Japanese [13, 14], Korean [15], and Taiwanese older adults [17]. A previous study suggests that body compositions are influenced by gender, age, and ethnicity [18] even among Asians [19]. Furthermore, AWGS 2019 recommendation did not consider the influence of body composition phenotype such as sarcopenic obesity. A recent study suggested that calf circumference adjusted with body mass index (BMI) should be used for patients outside the normal weight BMI range to eliminate the confounding effects of adiposity [20]. To date, there is scant data from Southeast Asian countries and no data is available for the Thai population. Our study aimed to evaluate calf circumference cutoff value in Thai independent older adults to define low muscle mass based on BIA and AGWS 2019 criteria.

## Materials and methods

### Study design and sample population

This cross-sectional study was conducted as a part of a geriatric cohort study at a university hospital in Bangkok, Thailand. The study recruited adults aged 60 years and older who visited the clinic for comprehensive geriatric assessments and health check-ups between March 2019 and May 2022.

The inclusion criteria required that participants were over 60 years of age and independent in their basic activities of daily living. The exclusion criteria included active malignancy or malignant diseases within 1 year of completed treatment, cardiac diseases, pulmonary diseases, or neurovascular diseases requiring hospitalization 3 months before enrollment, chronic renal diseases requiring renal replacement therapy, and unstable psychiatric disorders requiring medication adjustment 3 months before enrollment.

The institutional review board approved the cohort study (date of first registration on 25/10/2019, IRB approval no. 718/62). The study was registered with the clinical trial registry on 10/05/2020, with identifier number TCTR20200511003. Before participating in the study, all participants provided written informed consent. This study is reported following the STROBE guideline [21].

### Muscle mass and anthropometric measurement

To determine appendicular skeletal muscle mass (ASM), multi-frequency bioelectrical impedance analysis (BIA) was performed using an InBody770® machine (Inbody, Seoul, Korea). BIA has been proven to be reliable and interchangeable with dual energy X-ray absorptiometry (DXA) in diagnosing sarcopenia, according to previous studies [12]. The appendicular skeletal muscle mass index (ASMI) was calculated by dividing ASM by height squared. The 2019 criteria of the Asian Sarcopenia Working Group (AWGS) were used to identify low muscle mass, which was defined as < 7.0 kg/m^2^ in men and < 5.7 kg/m^2^ in women. Anthropometric data such as weight, height, calf circumference, and midarm circumference were also measured. Body Mass Index (BMI) was calculated by dividing weight by height squared. Calf circumference and midarm circumference were measured by determining the maximum value of both calves and mid proximal arms using a non-elastic tape while participants stood in a neutral position [15].

### Assessment of muscle strength

The handgrip strength of the participants was measured using a digital grip dynamometer (T.K.K.5401; Takei Scientific Instruments Co., Ltd., Niigata, Japan). The participants stood with their elbow fully extended and the maximum handgrip strength of two trials using the dominant hand was used [22].

### Assessment of physical performance

The usual gait speed of the participants was measured by having them walk 6 m at their natural pace. The average walking time of two trials was then converted into meters per second (m/s) [23].

### Statistical analysis

The characteristics of the subjected are presented by gender as mean ± standard deviation (SD). We compared the mean values of each characteristic between normal muscle mass and low muscle mass groups using Student’s *t-test*. Pearson’s correlation coefficients were performed to evaluate the correlation between calf circumferences or mid upper arm circumference and the BIA-measured ASM/height^2^. Receiver operating characteristic (ROC) analysis were performed to identify the optimal cut-off for calf circumference in screening LMM measured by BIA. We calculated the area under ROC curve, 95% confidence interval (95% CI), and the optimal cut-off point were determined using the shortest distance between ROC and upper left corner of the graph. Subgroup analysis of obese participants defined by BMI ≥ 25 kg/m^2^ for Asian population and older old age (age ≥ 75-year-old) were performed. Statistical significance was set as P < 0.05 in two-tailed tests. All statistical analyses were perform using SPSS statistics version 26 (IBM Corp., Armonk, NY, USA).

## Results

A total of 6,404 elderly adults, consisting of 78% women and 22% men, were enrolled. The mean age was 67.3 ± 5.1 years (Table 1). The prevalence of low muscle mass was found to be 47% in women and 25% in men. Elderly individuals with lower muscle mass were found to be statistically significantly older than those with normal muscle mass, with ages of 67.7 ± 5.3 years versus 66.5 ± 4.6 years in women (p < 0.001) and 70.3 ± 6.4 years versus 67.7 ± 5.3 years in men (p = 0.002). Additionally, elderly individuals with lower muscle mass had statistically significantly lower BMI and waist circumference in both women and men (p < 0.001) (Table 1). Among elderly men with low muscle mass, there was a statistically significant decrease in handgrip strength (p < 0.001) and slower gait speed (p < 0.001) compared to those with normal muscle mass. However, there was no statistically significant difference in gait speed between older women with low muscle mass and those with normal muscle mass. There was a statistically significant lower midarm (p < 0.001) and calf circumference (p < 0.001) both male and female elders in low muscle mass compared with normal muscle mass.

**Table 1:** Characteristics of study participants (N = 6,404)

	Women (*n* = 4959)			Men (*n* = 1445)		
Variables	Normal muscle mass	Low muscle mass^†^	*P*	Normal muscle mass	Low muscle mass^†^	*P*
n (%)	2648 (53)	2311 (47)		1086 (75)	359 (25)	
Age (Years)	66.5 ± 4.6	67.7 ± 5.3	< 0.001	67.7 ± 5.3	70.3 ± 6.4	0.002
ASM/height^2^ (kg/m^2^)	6.2 ± 0.5	5.2 ± 0.4	< 0.001	7.7 ± 0.5	6.6 ± 0.5	< 0.001
HGS (kg)	21.5 ± 3.5	19.3 ± 3.8	< 0.001	32.1 ± 5.8	27.5 ± 4.9	< 0.001
Gait speed (m/s)	1.44 ± 0.2	1.44 ± 0.2	0.662	1.54 ± 0.3	1.49 ± 0.2	< 0.001
Weight (kg)	60.8 ± 8.0	49.3 ± 5.5	< 0.001	70.8 ± 8.6	58.3 ± 7.1	< 0.001
Height (m)	155.1 ± 5.1	152.3 ± 5.0	< 0.001	167.4 ± 5.5	163.5 ± 5.5	< 0.001
Waist (cm)	84.3 ± 8.8	76.0 ± 7.7	< 0.001	90.5 ± 8.5	82.9 ± 8.0	< 0.001
BMI (kg/m^2^)	25.3 ± 3.4	21.3 ± 2.4	< 0.001	25.3 ± 2.8	21.9 ± 2.4	< 0.001
Midarm circumference (cm)	27.6 ± 3.1	24.5 ± 2.5	< 0.001	28.3 ± 2.8	25.3 ± 2.5	< 0.001
Calf circumference (cm)	35.3 ± 2.7	32.1 ± 2.3	< 0.001	36.9 ± 2.5	33.3 ± 2.3	< 0.001

Values were expressed as mean ± standard deviation or n (%)

^†^Low muscle mass was defined based on the Asian Working Group for Sarcopenia 2019 recommended cutoffs for ASM measured by BIA adjusted with height^2^ as follow: < 7.0 kg/m^2^ in male and < 5.7 kg/m^2^ in female. ASM, appendicular skeletal muscle mass; HGS, handgrip strength; BMI, body mass index

A total of 1,995 older adults had BMI ≥ 25 kg/m^2^ (Table 2) with a mean age of 67.2 ± 5.1 years. Among these, the mean ASMI for women was 6.35 ± 0.59 kg/m^2^ while for men was 7.85 ± 0.59 kg/m^2^. The prevalence of low muscle mass in obese older adults was 10% in women and 5% in men. There was a statistically significant decrease in gait speed among obese older women with low muscle mass compared to those with normal muscle mass. However, no significant difference in gait speed was observed between obese older men with low muscle mass and those with normal muscle mass. Moreover, both obese older women and men with low muscle mass exhibited a statistically significant reduction in midarm circumference (p < 0.001) and calf circumference (p < 0.001) compared to those with normal muscle mass.

**Table 2:** Characteristic of participants; subgroup-analysis of obesity (BMI ≥ 25 kg/m^2^) N = 1995

	Women (*n* = 1412)			Men (*n* = 583)		
Variables	Normal muscle mass	Low muscle mass	*P*	Normal muscle mass	Low muscle mass	*P*
n (%)	1271 (90)	141 (10)		554 (95)	29 (5)	
Age (years)	67.0 ± 4.9	69.9 ± 6.1	< 0.001	67.7 ± 5.3	71.1 ± 6.1	< 0.001
ASM/height^2^ (kg/m^2^)	6.5 ± 0.5	5.4 ± 0.3	< 0.001	7.6 ± 0.5	6.7 ± 0.2	< 0.001
HGS (kg)	21.2 ± 3.7	18.3 ± 3.3	< 0.001	32.1 ± 5.8	25.3 ± 4.9	< 0.001
Gait speed (m/s)	1.4 ± 0.2	1.3 ± 0.2	< 0.001	1.5 ± 0.3	1.4 ± 0.2	0.064
Weight (kg)	66.3 ± 4.2	57.9 ± 4.1	< 0.001	70.8 ± 8.6	68.2 ± 6.0	< 0.001
Height (m)	153.0 ± 5.1	148.4 ± 4.9	< 0.001	167.4 ± 5.5	160.2 ± 5.0	< 0.001
Waist (cm)	89.4 ± 8.2	86.0 ± 6.2	< 0.001	90.5 ± 8.5	94.8 ± 5.3	< 0.001
BMI (kg/m^2^)	28.0 ± 2.7	26.3 ± 1.1	< 0.001	27.3 ± 2.08	26.5 ± 1.2	< 0.001
Midarm circumference (cm)	27.6 ± 3.1	27.8 ± 2.3	< 0.001	28.3 ± 2.8	27.3 ± 1.7	< 0.001
Calf circumference (cm)	35.3 ± 2.7	**0.1**34.5 ± 2.4	< 0.001	36.9 ± 2.5	35.3 ± 1.6	< 0.001

Values were expressed as mean ± standard deviation or n (%)

ASMI, appendicular skeletal muscle mass index; HGS, Handgrip strength BMI, body mass index

A total of 585 participants in the study were 75 years old or older, with 66% being women and 38% being men. (Table 3) The average age of the group was 79.0 ± 3.5 years. Low muscle mass was found in 62% of women and 39% of men, with a mean ASMI of 5.49 ± 0.68 kg/m^2^ for women and 7.03 ± 0.82 kg/m^2^ for men. Statistically significant differences were observed in handgrip strength, BMI, midarm circumference, and calf circumference in both elderly women and men aged 75 years or older. Among male older adults aged 75 years or older, a statistically significant decrease in gait speed was found in those with low muscle mass compared to those with normal muscle mass. However, there was no statistically significant difference in gait speed among female older adults aged 75 years or older.

**Table 3:** Characteristic of participants; subgroup-analysis of older old adults (age ≥ 75 years) N = 585

	Women (*n* = 387)			Men (*n* = 198)		
Variables	Normal muscle mass	Low muscle mass	*P*	Normal muscle mass	Low muscle mass	*P*
n (%)	147 (38)	240 (62)		121 (61)	77 (39)	
Age (Years)	78.1 ± 2.6	78.9 ± 3.1	< 0.001	78.4 ± 3.0	79.9 ± 3.9	0.003
ASM/height^2^ (kg/m^2^)	6.2 ± 0.4	5.1 ± 0.4	< 0.001	7.5 ± 0.4	6.2 ± 0.6	< 0.001
HGS (kg)	19.4 ± 3.4	17.0 ± 3.4	< 0.001	28.6 ± 5.1	24.3 ± 4.7	< 0.001
Gait speed (m/s)	1.3 ± 0.3	1.2 ± 0.3	0.696	1.4 ± 0.3	1.3 ± 0.3	0.022
Weight (kg)	60.8 ± 7.0	49.8 ± 6.1	< 0.001	68.2 ± 6.7	56.9 ± 8.1	< 0.001
Height (m)	152.9 ± 5.1	150.8 ± 4.8	< 0.001	165.8 ± 5.4	161.8 ± 4.6	< 0.001
Waist (cm)	87.1 ± 9.2	79.1 ± 8.3	< 0.001	90.4 ± 7.6	84.3 ± 8.6	< 0.001
BMI (kg/m^2^)	26.0 ± 3.2	21.9 ± 2.7	< 0.001	24.8 ± 2.3	21.7 ± 2.6	< 0.001
Midarm circumference (cm)	27.9 ± 2.9	24.7 ± 3.0	< 0.001	27.4 ± 2.4	24.7 ± 2.6	< 0.001
Calf circumference (cm)	35.3 ± 2.7	**0.1** 31.9 ± 2.7	< 0.001	36.2 ± 2.1	32.4 ± 2.1	< 0.001

Values were expressed as mean ± standard deviation or n (%)

ASMI, appendicular skeletal muscle mass index; HGS, Handgrip strength BMI, body mass index

**Fig. 1:**
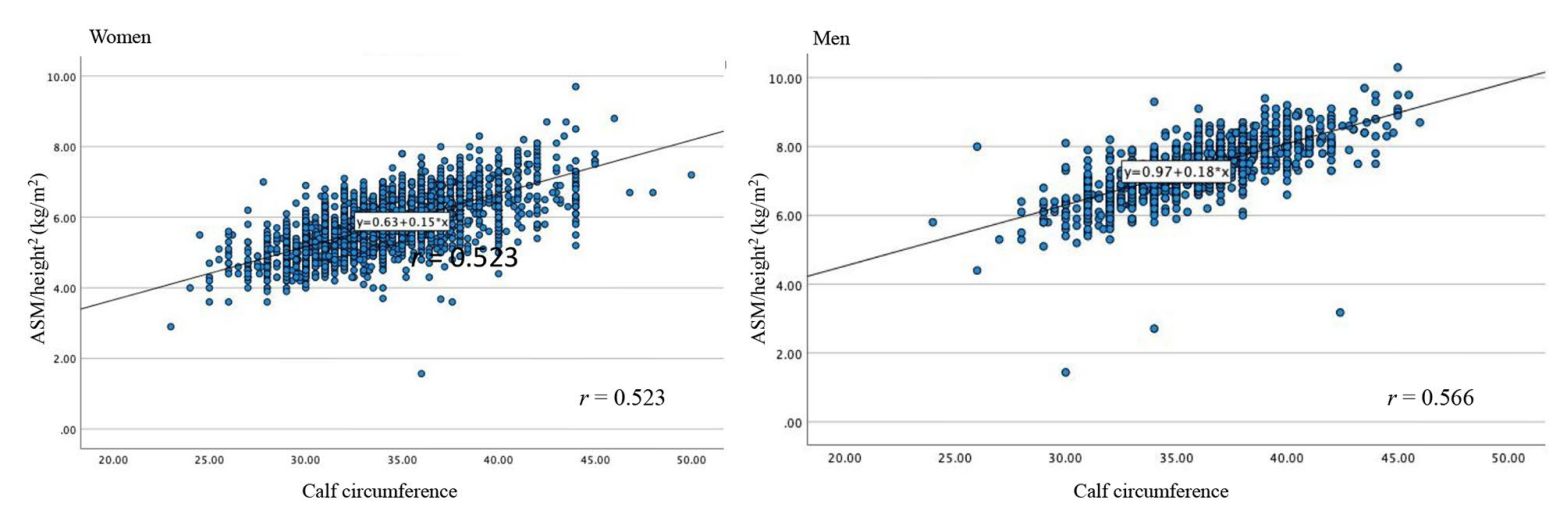
Correlation of calf circumference ASM/height^2^ in women and men. *r* = correlation coefficient. ASM, appendicular skeletal muscle mass

**Fig. 2:**
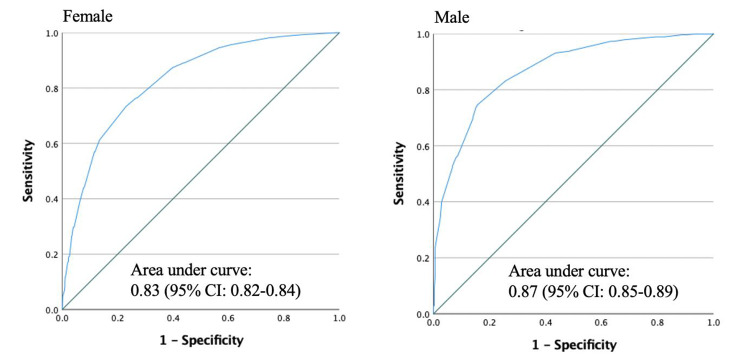
ROC curves for screening low muscle mass defined by BIA-measure ASM/height^2^ using calf circumference in men and women

ASM was positively correlated with calf circumference (men: r = 0.566, women r = 0.523; Fig. 1). The result of ROC analysis using calf circumferences values for screening low muscle mass is shown in Fig. 2.

The optimal cut-off values for women and men were < 33 cm (sensitivity 80.1%, specificity 60.5%) and < 34 cm (sensitivity 85.4%, specificity 70.2%), respectively (Table 4). When performing subgroup analysis of older adults with a BMI of ≥ 25 kg/m2, it was found that raising the cut-off point for calf circumference to < 34 cm (sensitivity 80.6%, specificity 54.0%) for women and < 35 cm (sensitivity 88.7%, specificity 55.2%) for men could increase specificity without substantially reducing sensitivity. Additionally, for older-old adult ≥ 75-year-old, the optimal cut-off values for women and men were < 33 cm (sensitivity 84.6%, specificity 79.9%) and < 34 cm (sensitivity 75.6%, specificity 87.0%), respectively.

**Table 4:** Optimal calf circumference cut-offs for screening low muscle mass

	Optimal calf circumference cut-off (cm)	Sensitivity (%)	Specificity (%)
• All older adults (n = 6,404)			
Men (n = 1445)	< 34	85.4	70.2
Women (n = 4959)	< 33	87.1	60.5
• Obese older adults (n = 1,995)			
Men (n = 583)	< 34	94.2	34.5
	< 35	88.7	55.2
Women (n = 1412)	< 33	89.5	33.6
	< 34	80.6	54.0
• Older adult aged ≥ 75 years (n = 1412)			
Men (n = 387)	< 34	75.6	87.0
Women (n = 198)	< 33	84.6	74.9

## Discussion

This study showed high prevalence of low muscle mass in independent Thai elderly adults (47% in women and 25% in men) and demonstrated a strong association between calf circumference and muscle mass, as determined through Bioelectrical Impedance Analysis (BIA), underscoring its potential as a screening tool for assessing the independence of older Thai adults. Additionally, our study suggests specific calf circumference cutoff values of 33 cm for women and 34 cm for men among the older Thai population. In the older-old adult subgroup (≥ 75 years), optimal cut-off values were the same cutoff values; <33 cm for women (sensitivity 84.6%, specificity 79.9%) and < 34 cm for men (sensitivity 75.6%, specificity 87.0%). Lower muscle mass significantly correlated with reduced BMI and waist circumference in both genders (p < 0.001). Subgroup analysis for those with BMI ≥ 25 kg/m2 suggested raising the cut-off for women to < 34 cm (sensitivity 80.6%, specificity 54.0%) and for men to < 35 cm (sensitivity 88.7%, specificity 55.2%) to enhance specificity without substantial sensitivity loss.

The influence of ethnicity on muscle mass is multifaceted, shaped by factors such as genetics, cultural practices, lifestyle, and dietary habits. However, it’s crucial to recognize that these influences can vary across populations, and broad generalizations may not universally apply to specific ethnic groups [24]. Cultural practices and lifestyle choices also contribute to variations in physical activity levels, exercise patterns, and occupational activities, all of which impact muscle mass. Moreover, diverse dietary patterns among ethnicities can affect nutrient intake, particularly protein consumption, a key factor in muscle development [25, 26]. Consequently, there is a need to validate calf circumference cutoff values across different ethnicities.

The recommendations from the Asian Working Group for Sarcopenia (AWGS) 2019 advocate calf circumference cutoff values of 33 cm for women and 34 cm for men in the older population, based on data from Japanese [13], Korean [15], and Taiwanese [17] older adult cohorts. Despite variations in Southeast Asian elderly populations, our study aligns with AWGS 2019 recommendations, suggesting their potential applicability and generalizability beyond East Asian populations to other Asian ethnicities and our findings endorse the practical use of these cutoff values in clinical settings within the region. The validated screening for low muscle mass is particularly crucial in middle-income countries like Thailand, classified as such by the World Bank [27], where financial considerations significantly impact healthcare decisions. Considering the budget constraints in these countries including low-income countries, calf circumference emerges as a favorable screening tool for identifying low muscle mass in settings with limited resources. Low muscle mass, particularly in older adults, can contribute to adverse health outcomes, including impaired physical function, an elevated risk of falls and fractures, and associations with metabolic abnormalities such as insulin resistance and impaired glucose tolerance. Moreover, it is linked to an increased risk of hospitalization and diminished overall quality of life [28–31].

In our subgroup analysis focusing on elderly individuals with obesity to determine the optimal cut-off point, we observed that increasing the threshold by 1 cm (34 cm in women and 35 cm in men) for older adults with a BMI ≥ 25 kg/m² resulted in higher specificity without significantly reducing sensitivity. This finding aligns with Gonzalez et al.‘s [20] conclusions, suggesting the need to adjust calf circumference values for BMI to mitigate potential confounding effects of adiposity. Notably, a higher BMI is typically associated with a larger calf circumference, attributed to either increased muscle mass or higher fat mass, particularly in postmenopausal women [29]. Therefore, using the AWGS-recommended calf circumference cut-off value in older adults with high fat-free mass poses a risk of underestimating low calf circumference prevalence and misidentifying individuals with low muscle mass among obese older adults. Based on our study results, we recommend employing a cut-off point of 35 cm for men and 34 cm for women in older adults with a BMI ≥ 25 kg/m² for an accurate assessment, acknowledging the impact of adiposity on this specific population.

Aging is linked to significant declines in muscle mass and strength, particularly among older-old adults (aged ≥ 75 years) [30]. A longitudinal study indicated a calf circumference decline of 1.1–3.4 cm over a 15-year period, with a more pronounced decrease observed in older-old adults compared to their younger counterparts [31]. Additionally, research has identified lower calf circumference cut-off values signifying low muscle mass in older adults with limited mobility, such as stroke patients (32 cm for women and 33 cm for men) [32], or those who have been hospitalized (28 cm for women and 30 cm for men) [33], in contrast to community-dwelling older adults. In our study, the cut-off value for calf circumference indicating low muscle mass in older-old adults (age ≥ 75 years) appeared similar to the overall results. Notably, the older adults in our cohort were functionally independent and physically active, suggesting that physical activity may have played a role in slowing the decline of calf circumference in the older-old group [34, 35]. Therefore, we recommend using the AWGS calf circumference cut-off value for functionally independent older adults, irrespective of age.

### Limitations of the study

Our study has limitations. Firstly, our participants were independent older adults, potentially restricting the generalizability of our findings to those with dependency, limited mobility, or frailty. Secondly, the cross-sectional design hinders our ability to establish a causal relationship between calf circumference, muscle mass decline, and the onset of sarcopenia. Cross-sectional studies offer a snapshot at a specific point, making it challenging to discern causality. Longitudinal studies tracking participants over an extended period would enhance our understanding of these relationships and provide more robust evidence. Acknowledging these limitations allows us to delineate the scope and relevance of our findings while identifying avenues for future research and exploration.

## Conclusion

Calf circumference proves to be a valuable screening tool for identifying low muscle mass in functionally independent older Thai adults, supporting the potential generalization of AWGS 2019 recommendations to the broader Asian elder population. Regardless of age, we recommend a threshold of 33 cm for women and 34 cm for men. For improved accuracy, it is advisable to raise the calf circumference cut-off value by 1 cm in obese older individuals, defined by a BMI ≥ 25 kg/m².

## Data Availability

The datasets analysed during the current study are available from the corresponding author on reasonable request.

## Abbreviations

DXA: Dual-energy X-ray absorptiometry
BIA: Bioelectrical impedance analysis
AWGS: Asian Working Group on Sarcopenia
ASMI: Appendicular skeletal muscle mass index
BMI: Body mass index
m/s: Meters per second
ROC: Receiver operating characteristic
